# Long-Term Exposure to Environmental Concentrations of the Pharmaceutical Ethynylestradiol Causes Reproductive Failure in Fish

**DOI:** 10.1289/ehp.7209

**Published:** 2004-11-04

**Authors:** Jon P. Nash, David E. Kime, Leo T. M. Van der Ven, Piet W. Wester, François Brion, Gerd Maack, Petra Stahlschmidt-Allner, Charles R. Tyler

**Affiliations:** ^1^Animal and Plant Sciences, University of Sheffield, Sheffield, United Kingdom; ^2^Laboratory of Aquatic Ecology, Katholieke Universiteit Leuven, Leuven, Belgium; ^3^School of Biological Sciences, University of Exeter, Exeter, United Kingdom; ^4^Rijksinstituut voor volksgezondheid en mileu (RIVM), Bilthoven, The Netherlands; ^5^L’Institut national de L’environnement et des risque (INERIS), Verneuil-en-Halatte, France; ^6^Hessisches Landesamt für umwelt und geologie (HLUG), Wiesbaden, Germany

**Keywords:** ecotoxicology, endocrine disruption, ethynylestradiol, mating systems, population effects, reproductive success, zebrafish

## Abstract

Heightened concern over endocrine-disrupting chemicals is driven by the hypothesis that they could reduce reproductive success and affect wildlife populations, but there is little evidence for this expectation. The pharmaceutical ethynylestradiol (EE_2_) is a potent endocrine modulator and is present in the aquatic environment at biologically active concentrations. To investigate impacts on reproductive success and mechanisms of disruption, we exposed breeding populations (*n* = 12) of zebrafish (*Danio rerio*) over multiple generations to environmentally relevant concentrations of EE_2_. Life-long exposure to 5 ng/L EE_2_ in the F_1_ generation caused a 56% reduction in fecundity and complete population failure with no fertilization. Conversely, the same level of exposure for up to 40 days in mature adults in the parental F_0_ generation had no impact on reproductive success. Infertility in the F_1_ generation after life-long exposure to 5 ng/L EE_2_ was due to disturbed sexual differentiation, with males having no functional testes and either undifferentiated or inter-sex gonads. These F_1_ males also showed a reduced vitellogenic response when compared with F_0_ males, indicating an acclimation to EE_2_ exposure. Depuration studies found only a partial recovery in reproductive capacity after 5 months. Significantly, even though the F_1_ males lacked functional testes, they showed male-pattern reproductive behavior, inducing the spawning act and competing with healthy males to disrupt fertilization. Endocrine disruption is therefore likely to affect breeding dynamics and reproductive success in group-spawning fish. Our findings raise major concerns about the population-level impacts for wildlife of long-term exposure to low concentrations of estrogenic endocrine disruptors.

Major worldwide attention has focused on the possibility that disruption of reproductive systems by endocrine-disrupting chemicals (EDCs) may be affecting the reproductive health of wildlife populations (Guillette and Gunderson 2001; Kime 1998; Tyler et al. 1998; Van Der Kraak 1998) and possibly, of humans (Colborn and Clement 1992; Ohtake et al. 2003). In fish, exposure to EDCs alters their reproductive physiology and morphology (Kime 1998; Tyler et al. 1998), resulting in, for example, the induction of female-specific proteins in male fish (Tyler and Routledge 1998), induction of gonopodia in females (Bortone and Davis 1994), reduced sperm counts (Haubruge et al. 2000), skewed sex ratios (Larsson et al. 2000), and prevalence of intersexuality (Jobling et al. 1998). Concern over the effects of EDCs on wildlife is driven by the hypothesis that disruption to the reproductive system may have serious deleterious consequences on the reproductive success of populations, but there is little evidence bearing on this expectation. An exception to this in a natural population is recent work on the roach (*Rutilus rutilus*); this study shows that sexual disruption (intersex) as a consequence of exposure to sewage treatment works effluents (STWs)—which contain a complex mixture of EDCs—results in gametes with reduced fertilizing capacity, as determined by *in vitro* studies (Jobling et al. 2002b). Direct population-level consequences of exposure to a specific EDC are known only for the antifouling agent tributyltin, which disrupts steroidogenesis, inducing an imposex condition that reduces reproductive success and causes localized extinctions in marine gastropods in the United Kingdom (Gibbs et al. 1991; Matthiessen and Gibbs 1998).

Many EDCs have a weak capacity to disrupt reproductive function. In contrast, natural steroidal estrogens control sexual differentiation and/or development in vertebrates and are potent modulators of sexual development and capacity (Bern 1992; Dawson 1998; Nagahama 1994; Strussmann and Nakamura 2002). Steroidal estrogens in effluents from STWs are believed to be responsible for, or contribute to, the feminized responses in some wild fish (Jobling et al. 1998, 2002a) and include the natural estrogens estradiol (E_2_) and estrone (E_1_) and the synthetic estrogen EE_2_, a component of the contraceptive pill (Desbrow et al. 1998; Tyler and Routledge 1998). In Europe, EE_2_ is present in effluents and surface waters at concentrations between 0.5 and 7 ng/L (Desbrow et al. 1998; Larsson et al. 1999; Ternes et al. 1999) but in some cases up to 50 ng/L (Ahern and Briggs 1989). A recent study of 139 streams in the United States found that 5.7% had concentrations > 5 ng/L (Kolpin et al. 2002a). In that study extremely high concentrations of EE_2_ up to 273 ng/L were reported at some riverine sites, but these figures may be overestimations and they are controversial (Kolpin et al. 2002b). EE_2_ concentrations are generally lower in surface waters than are natural steroidal estrogens, but the potency of EE_2_ in fish is 10- to 50-fold higher than that of E_2_ and E_1_ *in vivo* (Segner et al. 2003b; Thorpe et al. 2003) due to its longer half-life and tendency to bioconcentrate (650- and 10,000-fold in whole-body tissues and bile, respectively) (Lange et al. 2001; Larsson et al. 1999). In fish, for example, only 0.1 ng/L EE_2_ induces vitellogenin (VTG) yolk precursor (Purdom et al. 1994), 0.1–15 ng/L can affect normal sexual development and differentiation (Andersen et al. 2003; Metcalfe et al. 2001; van Aerle et al. 2002; Van den Belt et al. 2003; Weber et al. 2003), 2–10 ng/L can affect fecundity (Lange et al. 2001; Scholz and Gutzeit 2000; Van den Belt et al. 2002), 10 ng/L affects reproductive behavior (Balch et al. 2004), and 1–10 ng/L can reduce the fertilization success or viability of embryos from exposed adults (Hill and Janz 2003; Lange et al. 2001; Segner et al. 2003a). Thus, given its concentration in the environment, EE_2_ is potentially a major contributor to reproductive dysfunction in wild fish populations.

An overall aim of our research was to test whether there are any population-level consequences to reproductive dysfunction(s) induced by exposure to environmentally relevant levels of EDCs. Because of a number of compounding factors (e.g., larval dispersal, adult migration, etc.; Elliott et al. 2003) it is difficult to directly determine the population-level consequences of endocrine disruption on reproductive success in wild fish populations; even where it is possible to correlate levels of contamination with endocrine disruption and the consequential reproductive dysfunction with reproductive failure, this does not necessarily prove a direct cause-and-effect relationship. For these reasons, one approach to understanding the population-level consequences of reproductive dysfunction is to measure the impact of pollutants on reproductive success in model species bred under laboratory conditions (e.g., Balch et al. 2004; Hill and Janz 2003; Lange et al. 2001), an approach we have taken in our study. Reduced reproductive success may result from disruption of reproductive development, reduced female fecundity and male vitality, altered reproductive behavior, and/or disruption of the normal breeding dynamics. Little attention has been directed to the latter (Balch et al. 2004). To fully assess the potential of EDCs to disrupt reproduction, multigenerational full life-cycle exposures are needed that consider all relevant life stages and developmental end points; even where fish have been exposed over their whole life-cycle, the impact of any resulting reproductive dysfunction on their reproductive output has mostly been overlooked in earlier studies (Metcalfe et al. 2001; Van den Belt et al. 2003). A major goal of this study was to determine which stages or reproductive components are relatively most sensitive to endocrine disruption in terms of population-level impairment or failure to allow better priority and focus for future studies. We therefore determined impacts on reproductive success in fish populations over multiple generations and the mechanisms of reproductive impairment/failure investigated using environmentally relevant concentrations of EE_2_ and E_2_. Our chosen species for this work was the zebrafish because its short generation time facilitates the life-long and multigenerational chemical exposures; it is a group-spawner (a common breeding system in fish); and, most importantly, small populations will spawn naturally in the laboratory without any manipulation.

## Materials and Methods

### Fish culture and husbandry.

Wild-type zebrafish (*Danio rerio*, WIK strain; Max Plank Institute, Tubingen, Germany) maintained out-bred for three generations since capture were bred in our laboratory for one generation in clean freshwater before their use in this study. Adult fish were fed *ad libitum* twice daily on *Artemia* nauplii. Fry 5–12 days postfertilization (dpf) were fed a special fry diet and cultured rotifers, *Brachionus calciflorus*. *Artemia* nauplii were hatched in synthetic sea-water and rotifers reared in synthetic freshwater.

Populations were maintained and exposed to steroidal estrogens in 192 flow-through 18-L glass aquaria. An egg collection system allowed daily embryo collection without disturbance of the adult populations. Natural conditions were mimicked for optimal breeding; with a 13 hr light:11 hr dark photoperiod, artificial dawn/dusk, and water temperature of 28.5 ± 0.5°C (differences between tanks < 0.5°C throughout). Aquaria contained artificial weed for refuge and spawning substrate (glass marbles). Eggs were collected 1 hr after dawn. Embryos were maintained in 50 mL culture vessels modified for continuous flow-through before release into aquaria at 9 dpf.

### Water quality and chemical dosing.

Tap water was filtered with activated charcoal and reverse osmosis (RO; Osmonics E625 with cellulose membranes; GE Water and Process Technologies, Trevose, PA, USA). RO water was reconstituted with Analar grade mineral salts to standardized synthetic freshwater, to concur with U.S. Environmental Protection Agency (EPA) guidelines (U.S. EPA 1986). Water was aerated and heated to 28.5°C in a reservoir before it was supplied to each aquarium at a rate of 9 L/day to provide an exchange of 3 L/g biomass/day (U.S. EPA 1986). Aquarium water was continuously monitored for temperature, pH, and conductivity and routinely measured for carbonate hardness, oxygen, chlorine, iron, ammonia, nitrate, nitrite, and periodically for 142 pollutants; all were within acceptable limits of U.S. EPA guidelines (U.S. EPA 1986). The chemical (and control) aquaria were dosed independently at 20 mL/hr by a medical dripper system fed from a 2.5-L glass aspirator, and dosing rates were checked twice daily. 17α-Ethynylestradiol [17α-ethinyl-1,3,5(10)-estratriene-3,17β-diol] and estradiol [1,3,5(10)-estratriene-3,17β-diol] were initially dissolved in 100% ethanol at 1 mg/mL and serially diluted with sterile (treated with ultraviolet radiation) distilled water to a final stock concentration of 50 ng/mL. Fresh final stocks were made every 10 days and stored at 5°C. Aspirators were refilled every fourth day. Nominal estrogen doses were 0.5, 5, and 50 ng/L for EE_2_ and 5 ng/L for E_2_. The ethanol concentrations administered to aquaria were < 0.05 μL/L. Steroids were extracted from 5 L water samples for the EE_2_ (0.5 and 5 ng/L only, because the 50 ng/L treatment was terminated at an early phase of experimentation) and E_2_ (5 ng/L) using solid-phase extraction and measured by gas chromatography-mass spectrometry (Kelly 2000), with a detection limit of 0.1 ng/L.

### Impact of multigenerational estrogen exposure on reproductive success.

The main measures of reproductive success used in our study were the number of eggs produced by each population and the proportion of these eggs that were not viable at 14 hr post-fertilization (hpf; the mid-segmentation period) (Kimmel et al. 1995). The total reproductive success at 14 hpf was therefore the total number of viable embryos surviving to this stage. We used 14 hpf because infertile or dead embryos can be quickly differentiated from healthy embryos at this stage. Egg viability at 14 hpf was therefore a cumulative figure that includes the impact of unfertilized eggs plus embryo mortality during embryogenesis. Fertilization failure was differentiated from embryo mortality only in selected groups using time lapse videography to determine the mechanism(s) of reproductive failure.

At the begining of the study 720 adult (204 dpf) fish of mixed sex were randomly allocated to 60 aquaria (12 fish/aquarium) and acclimated for 14 days under egg collection conditions. Egg numbers and viability at 14 hpf were assessed in all groups in this F_0_ generation for 5 days before starting exposures. Tanks for the 12 replicates of the five treatments were randomly allocated using Latin square randomization in both vertical and horizontal gradients, and all experimentation was run blind of treatments. Steroid treatments were then initiated by flushing aquaria with 36 L estrogen-treated water 1 hr after spawning (day 0), and reproductive success was assessed over the following 15 days. The surviving F_1_ embryos were all reared to 100 hpf under continuous estrogen exposure, and the rate of embryo mortality/survival was assessed. We assessed the integrity of the surviving larvae, the proportion of embryos with developmental abnormalities, and the speed of development (proportion hatch and spine curvature) at 100 hpf as described by Kimmel et al. (1995). After the F_0_ generation had been exposed for a further 25 days (40 days of exposure total), we again assessed reproductive success (egg numbers and viability at 14 hpf), 100 hpf embryo mortality, and larval integrity of the resulting F_1_ offspring in eggs arising from 3 days of spawning. The surviving F_1_ embryos from these 3 days were then pooled within each replicate and reared to adulthood under continued exposure to form the F_1_ exposure generation. The age of the fish is stated as a single day postfertilization although, because the eggs were pooled over 3 days, the actual age may vary by up to an additional 48 hr. At 29 dpf, they were divided between two tanks (24 tanks/treatment) and maintained to 52 dpf, whereby survival and growth rates were determined. These fish were then pooled and randomly redistributed within treatments to provide 28 individuals/tank. At 72 and 124 dpf, populations were further reduced to 18 and 12 adults/tank, respectively. After the F_1_ generation had been exposed over their entire life time (210 dpf), their reproductive success was measured for 10 days. Any resulting F_2_ progeny were continually exposed to the estrogen treatments, and embryo survival/larval integrity was measured at 100 hpf.

### Analysis of estrogenic disruption and reproductive failure.

#### Life-stage sensitivity, transgenerational impacts, and recovery from estrogen exposure.

To determine which life stage(s) were primarily responsible for any reproductive failure and to test for any trans-generational effects, we exposed different subgroups to various regimes of noncontinuous estrogen exposure. Eggs were collected over a 4-day period from each treatment group in the F_0_ generation 16–19 days after the start of exposure (i.e., after the main egg collection period on days 0–15). In this first subgroup, the eggs from six of the 12 replicate tanks from each treatment (0.5 and 5 ng/L EE_2_, 5 ng/L E_2_ and control) were removed from the continuous treatment 1 hpf and reared to 100 hpf in clean water. Egg numbers and viability to 14 hpf and embryo survival/integrity to 100 hpf were compared between the F_0_ parent-only exposure, the continuous F_0_/F_1_ exposure, and the unexposed control group. To test for any transgenerational effects of parental exposure on adult reproductive integrity in their offspring, we set up a second subgroup in which the F_1_ generation was reared to adulthood in clean water. Eggs were collected from the F_0_ generation over 5 days from five replicate tanks in each treatment group (0.5 and 5 ng/L EE_2_, 5 ng/L E_2_ and control) 29 days after the start of exposure. These fish were then reared to adulthood, when end points of reproductive success, adult health, and embryo survival were measured at the same time as the main multigenerational exposure experiment, as described above.

To examine the ability of fish to recover from the effects of estrogen treatments on sexual differentiation and gonadal development, we established a third subgroup in which the F_1_ generation was removed from the treatments at 75 dpf and reared to adulthood in clean water. In this subgroup, we set up three replicate populations of mixed sex juveniles from each treatment (0.5 and 5 ng/L EE_2,_ 5 ng/L E_2_ and controls) using the excess fish removed from the main treatments at 75 dpf to reduce stocking densities; these fish were reared to adulthood in clean water. Reproductive success, adult health, and F_2_ embryo survival/integrity were measured in this subgroup at the same times as for the fish in the main multigenerational experiment.

#### Male replacement experiments.

To investigate the cause(s) of reproductive failure in the 5 ng/L EE_2_ treatment group after life-long exposure, males were removed from one-half of the replicate populations in the control group 20 days after embryo collection (240 dpf) to create an all-female group (*n* = 6) and leaving a mixed-sex control group (*n* = 6); we also replaced two EE_2_-exposed males with two control males in one-half of the replicate populations in the 5 ng/L EE_2_ exposure group to create two groups (*n* = 6), with or without additional healthy males. There was no dosing with EE_2_ during this phase of the experiment. The fish were acclimated, and egg numbers and egg viability were subsequently assessed over 5 days.

#### Adult health and growth.

In the F_0_ generation after 40 days of exposure, a total of 571 fish from 10 of the 12 replicates of each treatment were anesthetized, and weighed (wet weight, milligrams), and blood samples were collected from the caudal sinus. Gonads were dissected to determine gonadosomatic index (gonad weight as a percentage of body weight). Blood was centrifuged at 3,000 × *g* for 8 min and the hematocrit value measured in all samples. In the F_1_ generation (after full life-cycle exposure, 314 dpf), 284 adults from six replicate tanks in each of the remaining exposure groups (0.5 ng/L and 5 ng/L EE_2_ treatments with no F_1_ exposure; 0.5 and 5 ng/L EE_2_ and 5 ng/L E_2_ treatments with exposure stopped at 75 dpf in F _1_ ) were weighed; we then collected blood samples and determined the hematocrit value. Whole fish were fixed in Bouin’s fixative and embedded in paraffin, and the gonad region was sectioned to 4 μm, stained, and analyzed by light microscopy. Histologic analysis was conducted blind of treatment and was undertaken in three independent laboratories.

#### Fertilization success, sperm quality.

Time-lapse image capture was used to assess whether reductions in egg viability were due to reduced fertilization success or embryo mortality at 14 hpf. Digital images of four developing embryos were taken every 5 min 1 to 24 hpf and repeated for > 10 days per treatment. To assess sperm quality in F_1_ controls and 5 ng/L EE_2_ treatments only, males were stripped manually of expressible milt 1 hr before dawn, and the activated sperm was examined using video microscopy (Kime et al. 1996).

#### Plasma vitellogenin and steroids.

Whole-blood VTG concentrations were measured by enzyme-linked immunosorbent assay (ELISA), as described by Brion et al. (2002), in all 12 adults from four and three replicate tanks within each treatment sampled in the F_0_ and F_1_ generations, respectively. We assayed E_2_ and 11-ketotestosterone (11-KT) by ELISA (Nash et al. 2000) in 2 individuals from each of the eight replicate groups in each treatment (*n* = 8) for the F_0_ generation only.

### Statistical analyses.

Data were checked for normality using the Ryan-Joiner test and homogeneity of variance using Bartlett’s test. Data were transformed, where necessary, using square root (egg numbers and survival data), log_10_ (steroid levels), or arc sine of square root for proportion data (egg viability). When analyzing egg viability, we excluded data points for which egg numbers were < 4/tank (< 2%) because viability on low egg numbers biased the analysis disproportionately. We used analysis of variance (ANOVA) procedures except where there were unequal sample sizes and imbalance in design; then the GLM procedure was used. Post hoc analysis was performed against controls using the Dunnett’s test, and we used the Tukey test for between-treatment comparisons (male addition experiments). Data for egg numbers and viability to 14 hpf, survival and mortality of embryos to 100 hpf, and rate of embryo development were nested within the tank replicates to avoid pseudoreplication, and ANOVA was performed on 5-day means. When multiple measurements, such as weights and gonadosomatic index, were made from a single tank, these were also nested. VTG data did not conform to normality, so we adopted nonparametric analysis (Kruskal-Wallis). Significant deviations from expected sex ratios or levels of abnormal adult and gonadal morphology were tested using the chi-square test.

## Results and Discussion

### Natural variation in fecundity.

**Figure 1:**
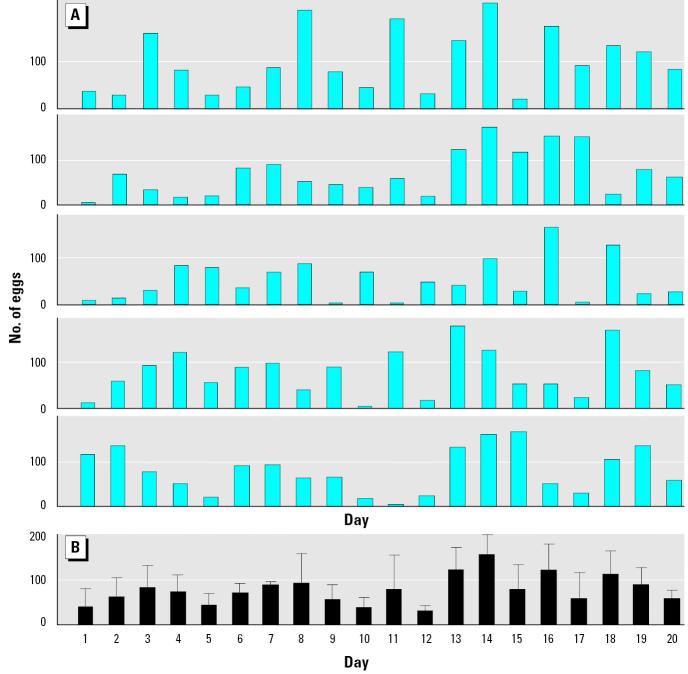
Between-tank and between-day variations in number of eggs over a 20-day period. (*A*) Total number of eggs in five random control tanks. (*B*) Mean (± SEM) number of eggs for the same tanks.

The average fecundity of this wild type strain of zebrafish was around 13 eggs/female, which is about 50% lower than that in some other inbred lines that have been selected for growth and reproductive output (Eaton and Farley 1974; Ensenbach and Nagel 1997). We found considerable variation in the numbers of eggs spawned daily in the zebrafish populations (Figure 1A,B), highlighting the need for extensive replication in studies of this nature. Our purpose, however, was to provide an experimental system that includes this natural variation found in wild populations. Cumulative and nested egg production was subsequently assessed over 5-day intervals to normalize measurements across the tanks; because females have a spawning periodicity of around 1.9 days (Eaton and Farley 1974), each female spawns at least once during a 5-day period. No significant differences in egg production occurred during the four consecutive 5-day periods in the F_0_ controls (mean, 0.2 eggs/female/day; *F* = 1.44, *p* = 0.27, *n* = 12, on 5-day nested means).

### Exposure concentrations of estrogens.

Measured mean concentrations of EE_2_ and E_2_ were between 90 and 100% of nominals: EE_2_ (mean ± SEM) concentrations were 0.5 ± 0.0 ng/L (0.5 ng/L EE_2_), 4.5 ± 0.3 ng/L (5 ng/L EE_2_), and E_2_ 4.8 ± 0.1 ng/L (5 ng/L EE _2_ ). EE _2_ and E _2_ were undetectable (< 0.1 ng/L) in the control group tanks and E_2_ was undetectable (< 0.1 ng/L) in the EE_2_ treatments. Mean ± SEM E_1_ concentrations in the control and treated tanks ranged between 0.5 ± 0.1 and 1.1 ± 0.1 ng/L and probably originated as an excreted product from the fish. There are no data on the effects of E_1_ in zebrafish, but in the rainbow trout, reproductive effects occur only at doses 3 orders of magnitude higher than the E_1_ concentrations found in the exposure aquaria (Thorpe et al. 2003). We used nominal steroid concentrations to describe the exposures in this study.

### Impacts of estrogen exposure on reproductive success during multigenerational exposure.

#### F_0_ generation.

For the 5-day period prior to the start of the estrogen exposures, we found no significant differences in egg numbers (*F* = 0.96, *p* = 0.43, *n* = 12), numbers of non-viable eggs at 14 hpf (*F* = 1.16, *p* = 0.34, *n* = 12), or level of mortality at 100 hpf (*F* = 0.91, *p* = 0.47, *n* = 12) between all five experimental groups (*n* = 12). There were also no differences in the reproductive output between the control groups in the F_0_ generation compared with the F_1_ generation, either in egg numbers (*F* = 0.52, *p* = 0.47, *n* = 12), nonviable eggs at 14 hpf (*F* = 2.8, *p* = 0.11, *n* = 12), or embryo mortality at 100 hpf (*F* = 0.45, *p* = 0.51, *n* = 12) for pooled 15-and 10-day means in each generation. A mean of 91.5% of the eggs were fertilized and survived to 100 hpf (posthatch) in all treatments before exposure and in control treatments throughout the experiment. This level of fertilization and survival is high when compared to similar studies on zebrafish (Hill and Janz 2003), sheepshead minnows (*Cyprinodon variegatus*; Zillioux et al. 2001), and medaka (*Oryzias latipes*; Balch et al. 2004), where lower survival (70, 65, and 62%, respectively) in controls probably relates to suboptimal breeding conditions or stresses associated with embryo culture.

**Figure 2:**
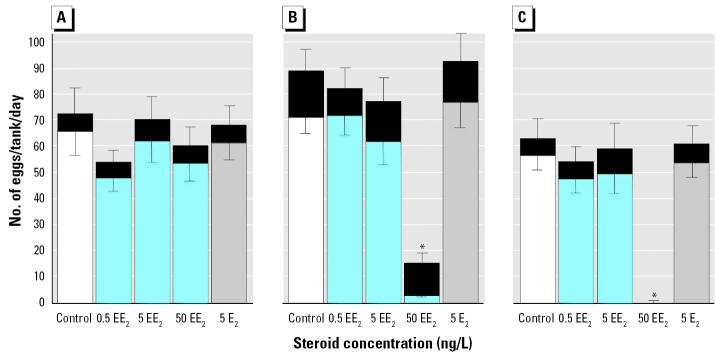
Reproductive success in the F_0_ generation of zebrafish exposed to 0.5, 5, and 50 ng/L EE_2_, 5 ng/L E_2_, and unexposed controls for three consecutive 5-day periods: (*A*) 1–5 days, (*B*) 6–10 days, and (*C*) 11–15 days. Total bar length indicates the total number of eggs per tank (mean ± SEM; top error bar). The lighter bar indicates total survival of viable eggs at 14 hpf (mean ± SEM; bottom error bar). The black section indicates the number of nonviable eggs at 14 hpf.
*Significant decrease in egg number and 14 hpf viability when compared with the control group for each period, and increase in rate of nonviable eggs when compared with the same group in (*A*); ANOVA, all cases *n* = 12, *p* < 0.01, post hoc analysis against control treatments by Dunnet’s test with *p* > 0.05.

The short term exposure to 50 ng/L EE_2_ in the F_0_ generation caused a time-related reduction in egg production and egg viability to 14 hpf (Figure 2; two-way ANOVA for all cases: *n* = 12, *p* < 0.01) and no survival of their F_1_ offspring to 100 hpf. After 10 days exposure there was complete reproductive failure (no egg production) in the 50 ng/L EE_2_ exposure group. These data support previous findings for high dosage, short-term effects of EE_2_ (Lange et al. 2001; Scholz and Gutzeit 2000; Seki et al. 2002; Van den Belt et al. 2002; Zillioux et al. 2001), and this treatment was subsequently terminated. There were no effects, however, of any other estrogen treatment on numbers and viability of eggs from the F_0_ generation at 14 hpf (Figure 2), embryo mortality in the F_1_ generation (*F* = 0.36, *p* = 0.78, *n* = 12), or impacts on larval integrity at 100 hpf; the proportion of developmental abnormality, hatch rate, and level of spine curvature were all not significantly different from controls; *p* > 0.05 for all cases. Similarly, we found no effects after an additional 26-day exposure (40 days continuous exposure) to 0.5 ng or 5 ng/L EE_2_ or 5 ng/L E_2_ on egg production (*F* = 1.31, *p* = 0.28, *n* = 12), egg nonviability (*F* = 1.30, *p* = 0.28, *n* = 12), or F_1_ mortality to 100 hpf (*F* = 1.71, *p* = 0.18, *n* = 12) and larval integrity (*p* > 0.05 for all cases) at 100 hpf.

Mean cumulative survival at 52 dpf was between 66% and 79%, with no differences (*F* = 1.1, *p* = 0.35, *n* = 12) between the treatments. This rate of survival is high for cultured zebrafish embryos (Ensenbach and Nagel 1997) and was higher than a comparable study (50%; Hill and Janz 2003). Most (98%) of this mortality occurred during the first stages of exogenous feeding and was not related to treatment. There were no effects of the estrogen treatments on growth at 52 dpf (mean = 65–80 mg, *F* = 1.45 *p* > 0.24, *n* = 12). The mortality rate between 52 dpf and 7 months of age was < 0.2% throughout, and we found no differences (*F* = 0.65, *p* > 0.63, *n* = 12) in fish weight between treatments for either sex at the end of the experiment when fish were slaughtered (9 months; mean = 232–349 mg).

#### F_1_ generation.

**Figure 3:**
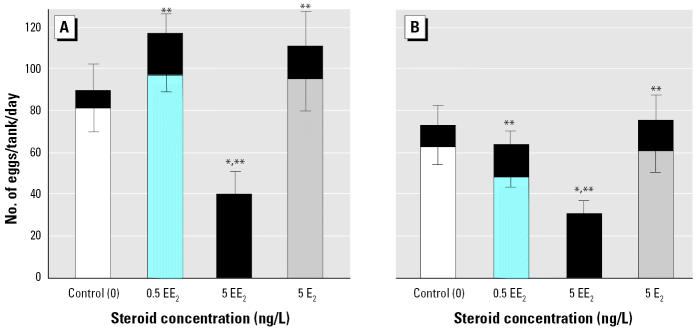
Reproductive success of the F_1_ generation of zebrafish after 7 months (210 dpf) exposure to 0.5 and 5 ng/L EE_2_, 5 ng/L E_2_, and unexposed controls for two successive 5-day periods: (*A*) 1*–*5 days, and (*B*) 6–10 days. Total bar length indicates the total number of eggs per tank (mean ± SEM; top error bar). The lighter bar indicates the total number of viable eggs at 14 hpf (mean ± SEM; bottom error bar). The black section indicates the proportion of eggs nonviable at 14 hpf.
*Long-term exposure to 5 ng/L EE_2_ resulted in a reduced fecundity (*n* = 12, *p* < 0.01) and no survival past 14 hpf. **The proportion of nonviable eggs was significantly higher for all treatments when compared with the control rates (*n* = 12, *p* < 0.05).

In complete contrast, life-long exposure (210 dpf) to 5 ng/L EE_2_ resulted in complete reproductive failure in the F_1_ generation, with no viability in the eggs at 14 hpf (Figure 3); we found no viable eggs in almost 12,000 spawned (*F* = 7.6, *p* < 0.001). Egg production was also reduced in fish in the 5–ng/L EE_2_ exposure group, approximately 42–45% of that of the control for the two successive 5-day assessment periods (Figure 3; *F* = 207, *p* < 0.001). We found no effects of either 0.5 ng/L EE_2_ or 5 ng/L E_2_ on egg numbers, but proportions of nonviable eggs/total eggs spawned at 14 hpf in both of these treatments were more than twice that in the controls (*F* = 207, *p* < 0.001; post hoc comparisons with *p* < 0.05). A large variation in egg numbers, however, meant that these effects on viability at 14 hpf did not affect total reproductive success at 14 hpf (i.e., total number surviving 14 hpf) in the 0.5 ng/L EE_2_ or 5 ng/L E_2_ treatments (Figure 2). The rate of embryo mortality to 100 hpf in the surviving F_2_ embryos was low (< 1%) and not significantly increased by these low exposures (*F* = 0.63, *p* < 0.48, *n* = 12). Larval integrity (proportion of developmental abnormality, hatch rate, and level of spine curvature) of the F_2_ embryos at 100 hpf in these surviving groups was also not affected.

Two recent studies have also examined the impact of full life-long exposure to EE_2_ on reproductive success in other fishes. Lange et al. (2001) found that life-long exposure to 0.2 and 1 ng/L EE_2_ in the fathead minnow (*Pimephales promelas*) caused 20 and 35% reductions, respectively, in the offspring’s hatching success and no impact on fecundity, which was comparable with the impact of our 0.5 ng/L EE_2_ exposure. In the Lange et al. study, however, the impact on reproductive success of a higher dose (4 ng/L EE_2_), one similar to that which would have caused complete reproductive failure in our study (5 ng/L EE_2_), was not tested because it was not possible to sex these fish for the pair-wise breeding setup used. In a study on medaka, Balch et al. (2004) found no significant effects of life-long exposure at lower doses (0.2 and 1 ng/L EE_2_), but at 10 ng/L EE_2_ (there was no intermediate dose) they found complete reproductive failure, which was related to suppressed reproductive activity.

Thus, life-long exposures to very low and environmentally relevant concentrations of EE_2_ have severe and deleterious effects on reproductive success for breeding populations of zebrafish, and there is evidence that these strong effects will occur in other species at similar concentrations (Balch et al. 2004; Lange et al. 2001). Furthermore, these effects occur at concentrations that are at least an order of magnitude lower than for short-term exposures of mature fish proximate to spawning time; Seki et al. (2002), Van den Belt et al. (2002), and Zillioux et al. (2001) provide other examples of lower sensitivity to adult-only exposure.

### Analysis of endocrine disruption.

#### Reproductive failure in F_0_ generation.

We found that 50 ng/L EE_2_ was acutely toxic and resulted in 35% mortality; in the surviving fish there were negative effects on a wide range of health measures, including reduced hematocrit, increased spinal deformities, and reduced gonad growth. Reproductive failure ensued because of complete cessation of spawning in this treatment group (Denslow et al. 1999; Seki et al. 2002; Van den Belt et al. 2002). There was no reduction in reproductive success or health effects in any of the other estrogen treatment groups.

#### Reproductive failure in F_1_ generation.

**Figure 4:**
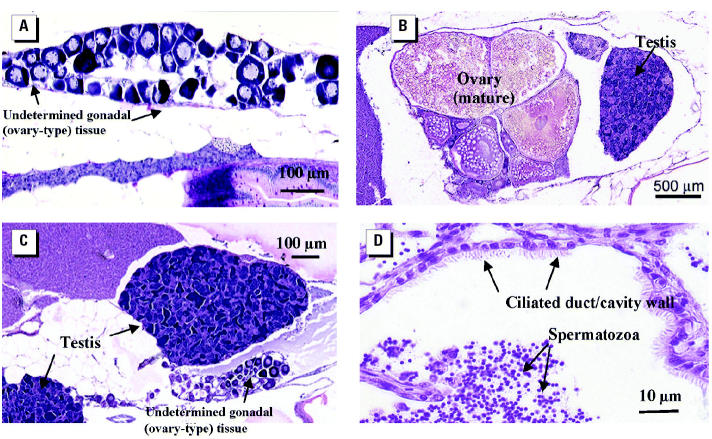
Effects of life-long exposure to 5 ng/L EE_2_ on gonad development in adult zebrafish (314 dpf). (*A*) Persisting juvenile undifferentiated (ovary-type) gonad in presumptive males. (*B*) Intersex fish with one ovary and one testis. (*C*) Intersex fish with two testes and smaller juvenile (ovary-type) tissue. (*D*) Ciliated sperm duct in testis of mature male (found only in adults when exposure to EE_2_ was stopped at 75 dpf).

We investigated the mechanism(s) of disruption leading to reproductive failure in the F_1_ generation by assessing sperm quality, fertilization success in their offspring (F_2_ generation), gonad development and maturation, and male reproductive behavior in the breeding populations. In the 5 ng/L EE_2_ treatment group, we found no phenotypic males, as discerned by the absence of any secondary sex characteristics, such as slightly yellow/bronze coloration and bright anal fin markings. This gave the initial impression that sex reversal had been induced, as occurs in some fish species after exposure to steroidal estrogens (Iwamatsu 1999; Lange et al. 2001; Scholz and Gutzeit 2000). Further studies on these F_1_ fish found that no fish contained expressible sperm. The hypothesis that reduced egg viability at 14 hpf was due to nonfertilization rather than early embryo mortality was confirmed through hourly time lapse image analyses of egg/embryo development for this treatment. Gonadal histology on the F_1_ fish after life-long exposure to 5 ng/L EE_2_ established that none of the males had normal testes. Of these fish, 43% had gonads that had not fully differentiated into testes; these undetermined gonads resembled primary stage ovary-type tissue, which is the natural condition of immature fish during early stages of normal male gonadal differentiation (Maack and Segner 2003). Figure 4A shows an example. Gonadal evidence that these fish were indeed feminized males was further supported by the concentrations of blood VTG in these animals. Via histology, we found that all of these life-long exposed fish that were not clearly mature/maturing females had compromised gonads and also had very low concentrations of blood VTG (mean ± SEM = 1.8 ± 1.1 μg/mL), whereas fish that were definitively females contained extremely high concentrations of blood VTG (1,092 ± 106 μg/mL). We found no fish with an intermediate response. This highly dichotomous response to the estrogenic treatment after long-term exposure, which was strongly correlated to the histology and behavior—these dysfunctional males showed natural spawning behavior—gives good evidence these differential responses were determined by the underlying genetic sex. The interpretation that long-term exposure to estrogens suppresses male pattern sexual differentiation and arrests testes development rather than producing functional females is confirmed by earlier work on zebrafish (Hill and Janz 2003; Segner et al. 2003a; Van den Belt et al. 2003; Weber et al. 2003). In some of these earlier studies on zebrafish (Hill and Janz 2003; Weber et al. 2003), males with undifferentiated testes have been categorized as females and have been reported as a skewed sex ratio. This is a slightly misleading interpretation in an undifferentiated gonochorist species such as the zebrafish, because males that have arrested sexual differentiation, while superficially resembling early ovary type tissue (as do both sexes at this stage), do not develop into functional females and their gonads will differentiate into testes when removed from exposure (Hill and Janz 2003). It would be more accurate to describe these fish as simply showing undetermined gonadal sex rather than assigning female status.

We found a low incidence of intersex gonads (four fish; Figure 4B,C) in the 5 ng/L exposure group. Although similarly low occurrences of intersex have been found in various species when exposed to estrogens during development (Van den Belt et al. 2003), the high level of intersex found in some natural species (Jobling et al. 1998) has not been replicated in the laboratory. This may relate to between-species differences in their susceptibility to this intersex condition or it could indicate the involvement of other, as yet, unknown chemical or environmental factors. It is doubtful if the fish with intersex gonads in our study were sexually functional because they also had extensive malformations of the ovarian and sperm ducts, as shown in other species [e.g., carp (Gimeno and Komen 1996) and fathead minnow (van Aerle et al. 2002)]; there was no fertilization in these groups. Further data on the effects on the histopathology and enhanced images are available online in the “Toxicological Pathology Atlas of Small Laboratory Fish” (RIVM 2004).

### Behavioral analysis and male replacement experiments.

**Figure 5:**
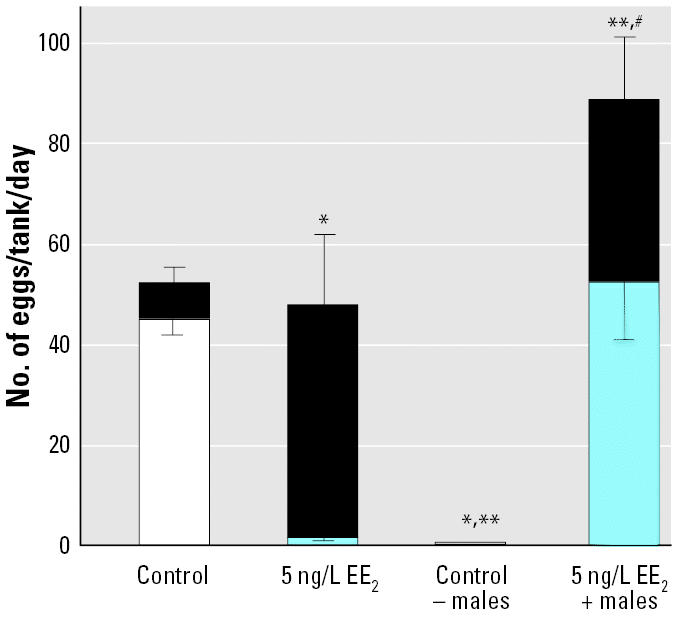
Reproductive success (5-day means) in the F_1_ generation of zebrafish at 240 dpf after lifelong exposure to 5 ng/L EE_2_, with subsequent manipulation of males in the populations (all experiments done under no direct exposure). In the controls, males were either retained (control; six tanks) or removed (control – males; six tanks). In the EE_2_ treatment, EE_2_-exposed males were either retained (5 ng/L EE_2_, six tanks) or two males were substituted with two healthy control males (EE_2_ + males; six tanks). Total bar length indicates the total number of eggs per tank (mean ± SEM) for the six replicate tanks in each group. The lighter bar indicates the total number of viable eggs (mean ± SEM) to 14 hpf. The black section indicates the percentage of nonviable eggs at 14 hpf.
*Significantly different (*p* < 0.05) from control in the proportion of viable eggs at 14 hpf. **Significantly different (*p* < 0.05) from control in total egg numbers. ^#^Significantly different (*p* < 0.05) number of eggs laid with the addition of healthy males compared with controls (*F* = 16.5, *p* < 0.001, *n* = 6); the proportion of nonviable eggs was still significantly higher (*F* = 177, *p* < 0.001, *n* = 6) than in the control group.

Close observation of the F_1_ adults in the 5 ng/L EE_2_ treatment group indicated that even though there was no fertilization, natural spawning behavior still occurred and resulted in egg-laying activity, even though these fish were all superficially female. Removing all the males from control tanks in the F_1_ generation caused complete cessation of spawning, showing that the presence of males, or at least male pattern behavior, was needed to stimulate spawning in females (Figure 5).

When two healthy nonexposed males were taken from the control group and substituted for two males from populations previously exposed to 5 ng/L EE_2_ (six tanks), there was an increase in embryo viability, showing that the females in the 5 ng/L EE_2_ group were fertile. Even with healthy males, the rate of survival to 14 hpf was, however, significantly less than in the control group. This reduction in survival to 14 hpf was due to reduced fertilization success (tested using time-lapse videography) and indicates that the effects were not due to reduced egg quality in the EE_2_-exposed females. Experiments conducted in our laboratory have shown that even when the population sex ratio is strongly biased toward females in normal healthy populations (14 females:2 males), fertilization rates are > 90%. Thus, the high level of unfertilized eggs in the 5 ng/L EE_2_ exposures with replacement males is unlikely to be a function of the presence of only two fertile males. Close observations of the spawning activity in these tanks revealed that sexually compromised males actively participated in the spawning act, chasing females and competing with the healthy males for proximity to the females as they spawned. Thus, it was clear that the reduced fertilization was, at least in part, due to infertile males interfering with the fertilization capability of healthy males. Egg production in the EE_2_-treated group with healthy males was higher when compared with controls (Figure 5). The presence of vigorous, healthy males may have induced the greater egg production in these females after a prolonged period with EE_2_-exposed males. Moreover, egg production in this treatment group was similar to previous controls; egg production in the associated controls was relatively low.

The data from this experiment show that normal gonadal differentiation and development is relatively more sensitive to disruption than is male reproductive behavior. We suggest that there is a higher threshold on sensitivity to behavioral disruption relative to exposure concentrations that cause inhibition of functional testes development. Although few studies examine the impact of endocrine disruption on reproductive behavior in relation to reproduction success, Balch et al. (2004) confirmed that medaka behavior was unaffected at doses of EE_2_ < 10 ng/L, even though there were significant gonadal abnormalities at exposures of 2 ng/L.

Retention of a normal pattern of behavior in the infertile males that affects the fitness of other males may have an even greater impact on the reproductive success than if these males did not participate in spawning. The findings illustrate that information on the effects of EDCs and the interactions between fish within a spawning group (i.e., their mating systems) is necessary to develop our understanding of population-level implications of endocrine disruption. In the wild, group-spawning fish come together on a spawning ground; they may have migrated from different areas, and therefore different chemical exposure regimes, and thus they may have different degrees of disruption to their reproductive systems. To date, none of these factors has been taken into consideration when evaluating the potential impacts of EDCs such as EE_2_ on breeding success in wild fish populations.

#### Transgenerational effects, life stage sensitivity, and recovery from EE_2_ exposure.

It is well documented that maternal transfer of the synthetic estrogen diethylstilbestrol into the developing fetus in humans resulted in cases of sexual dysfunction, which became manifest at puberty (Bern 1992). Maternal transfer of EDCs may also occur in fish, when pollutants are co-transported with VTG into the developing oocyte (Gray et al. 1999). In the present study we found no transgenerational effects of F_0_ exposure to < 5 ng/L EE_2_ on the level of embryo viability at 14 hpf or F_1_ embryo mortality and larval integrity (proportion of developmental abnormality, hatch rate, and level of spine curvature) at 100 hpf, when the offspring were reared in clean water and compared with the corresponding treatment in continuous F_0_ and F_1_ exposure or control treatment (*n* = 6, *p* > 0.05 in all cases). This is not surprising because we also found no effects up to 100 hpf when both the parents (F_0_) and offspring (F_1_) were exposed at these same concentrations during the main multigenerational experiment. Moreover, reproductive success in the F_1_ subgroups (*n* = 5) reared to adulthood in clean water but with parental F_0_ exposure (0.5 and 5 ng/mL EE_2_ and 5 ng/mL E_2_) was not affected, either in the number of eggs spawned by the F_1_ (*F* = 1.07, *p* = 0.39 *n* = 6) or the 14 hr viability of the resulting eggs (*F* = 0.84, *p* = 0.29; six tanks per treatment) when compared to the control eggs collected at the same time. This contrasts with continuous multigenerational exposures (F_0_ and F_1_) causing significant or complete reproductive failure at similar doses. F_0_-only exposure also had no consequences for the integrity of the F_2_ offspring, either in the level of embryo mortality or larval integrity at 100 hpf (*p* > 0.05 in all cases), again consistent with what would be expected if there were no transgenerational impact. These results, therefore, give little evidence of any transgenerational (maternal) effects of exposure to EE_2_ or E_2_, at least when measuring population level consequences for reproductive success and survival. There are no other studies that have reported transgenerational effects of steroid hormones on reproductive success, although a study by Foran et al. (2002) showed transgenerational impacts on VTG and estrogen receptor responsiveness in medaka, but only at extremely high doses (> 500 ng/L). Reported transgenerational impacts of other endocrine disruptors, such as DDT on F_1_ gonadal development (Metcalfe et al. 2000) and nonylphenol on F_1_ steroid levels (Schwaiger et al. 2002), may be related to different biochemical properties of these pollutants.

**Figure 6:**
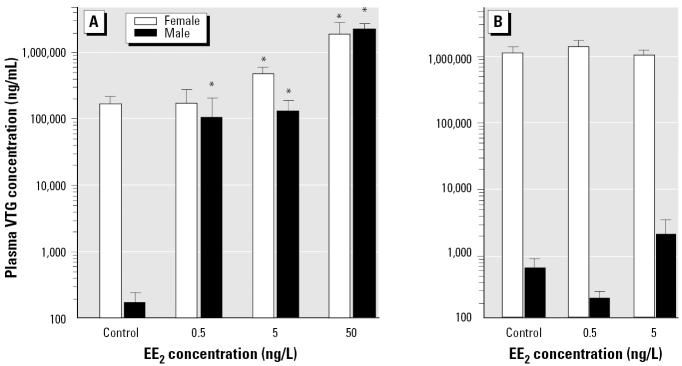
Whole blood VTG concentrations (mean ± SEM) in male and female zebrafish in the F_0_ generation after 40 days exposure to 0.5, 0.5, 5, or 50 ng/L EE_2_ (*A*) and in the F_1_ generation after life-long (310 dpf) exposure to 0, 0.5, or 5 ng/L EE_2_.
*Dose-dependent induction of VTG (*p* < 0.05 compared with controls of the same sex).

In populations (three tanks) of fish exposed to 0.5 and 5 ng/L EE_2_ and 5 ng/L E_2_ to 75 dpf and then reared in clean freshwater to adulthood (a depuration of 5 months), we found no differences in fecundity at 210–220 dpf when compared with the control for these subgroups (*F* = 0.46 *p* = 0.72, 10-day means). This is in contrast with the strong impact of continuous full life-long exposure (Figure 6) to 5 ng/L EE_2_ in the F_1_ generation that caused a considerable reduction in egg numbers, thus illustrating a capacity for recovery in their reproductive output (numbers of eggs spawned).

Interestingly, however, even after a 5 month period of depuration, there was still a highly significant (*F* = 12.81 *p* = 0.003) impact of exposure on fertilization success; the proportion of nonviable eggs at 14 hpf increased to 16.2% in 0.5 ng/L EE_2_ and 24% in 5 ng/L EE_2_ compared with a rate of 7.8% in the controls. Even though males were still able to produce sperm (shown by stripping) and fertilize the eggs spawned by females, there was also persistent disruption to testes development, which could explain the reduced fertilization success. Histology showed extensive malformations of the sperm ducts, the presence of an ovarian cavity in the testis, variation in proportion of testicular cell types, and ciliation of sperm ducts (Figure 4D), a feature normally found in ovarian ducts that has been reported in wild intersex roach (Nolan et al. 2001). Persistent effects on testes of developmental exposure to similar levels of EE_2_ in zebrafish were not reported by Weber et al. (2003), but duct morphology was not examined. Partial recovery from EE_2_ exposure during early sexual differentiation can therefore occur with a sufficient period of depuration, but certain morphologic effects are long lived. The persistent nature of EDC-induced gonadal abnormalities has been confirmed in several fishes (Gimeno and Komen 1996; McAllister and Kime 2003; Scholz and Gutzeit 2000; van Aerle et al. 2002), and differences in the level of recovery after depuration probably relate to diverse modes of sexual differentiation found between species.

#### Vitellogenin and steroid hormone response.

In the F_0_ generation after 40 days exposure to the lower, sublethal concentrations of EE_2_, we found a highly significant (*F* = 41.64, *p* < 0.001) and dose-related induction of VTG in both sexes (Figure 6A). In males, a dose of only 0.5 ng/L EE_2_ induced a 5,000-fold increase in blood VTG, which concurs with previous studies in fish, including zebrafish (Fenske et al. 2001; Thorpe et al. 2003). There was also a significant (*F* = 17.59, *p* < 0.001, *n* = 8) and dose-related suppression of the major male fish sex hormone 11-KT in F_0_ males; in the blood of fish treated with 0.5, 5, or 50 ng/L EE_2_ or 5 ng/L E_2_, 11-KT concentrations were 30, 5, 6, and 8% of controls, respectively (mean ± SEM = 94 ± 46 ng/mL). The sensitivity of both VTG and 11-KT to exogenous steroidal estrogens reinforces their value as biomarkers for exposure to estrogen after short-term exposure (Brion et al. 2002; Denslow et al. 1999). Very considerable (3 orders of magnitude) changes in blood VTG and up to 95% reduction in 11-KT, however, did not impact the short-term reproductive success of the zebrafish populations, emphasizing their utility as biomarkers of estrogen exposure, rather than necessarily measures of reproductive impact. There were no significant impacts of the estrogen treatments on endogenous blood E_2_, although there was a high level of variability between fish. The measurements of 11-KT and E_2_ are the first data on reproductive steroid hormones in zebrafish, and the method (Nash et al. 2000) could be adapted to other small species (≤300 mg). To further investigate natural levels of a range of steroids in zebrafish and their response to endocrine disruption, ongoing research in our laboratories is examining a larger number of replicates that were collected in this study and in new experiments.

In contrast with the short-term adult exposure (Figure 6A), we observed no induction of VTG in males after life-long exposure to < 5 ng/L EE_2_ (Figure 6B), suggesting an acclimation to the EE_2_ exposure. Similarly there were no effects of a multigenerational life-long exposure to estrogen on VTG in females, but the VTG concentration in F_1_ controls was higher than in both the F_0_ controls and 5 ng/L EE_2_ fish (Figure 6B). It seems likely that there is strong down-regulation of vitellogenic response after long-term exposure to exogenous estrogens, which has not been reported before. Studies in fish have shown that there are seasonal variations in the number of estrogen receptors and in the ligand affinity of these receptors (Smith and Thomas 1991), and thus responsiveness to estrogen, but we do not know the mechanism for acclimation to estrogen observed here in males. A reduced vitellogenic response (or indeed lack of a response) in males after long-term exposure to low levels of estrogen further complicates the use of VTG as a biomarker for estrogen exposure in wild populations; fish that have been subjected to long-term exposure to estrogen and with no measurable blood VTG could still be reproductively compromised.

The temporal disassociation between the impact of estrogen exposure on reproductive success and the vitellogenic response also clearly shows that the perturbations to reproductive physiology that ultimately cause reproductive failure are probably not directly linked or at least not a direct consequence of the stimulation of vitellogenin.

## Conclusions

Overall, our findings on the effective concentration of EE_2_ on reproductive success raise serious concerns for possible population-level effects on fisheries. Concern about population-level consequences associated with exposure to EE_2_ in the aquatic environment is further exacerbated by the fact that detection capabilities for routine assessments of EE_2_ in effluents and environmental samples are generally above concentrations that are biologically significant (Kolpin et al. 2002a). Extremely high potency and persistence in the aquatic environment (Johnson and Williams 2004) is also likely to be mirrored by other persistent pharmaceuticals which are, as yet, not measured in routine assessments of effluents (Metcalfe et al. 2003).

The data presented in this article give evidence that different reproductive components have differential sensitivities to endocrine disruption and that these sensitivities are dependent on the length of exposure and timing of exposure relative to development and maturity. Full life-long exposures had a strong impact on reproductive success at a concentration that was at least one order of magnitude less than when fish were given short-term exposures proximate to spawning. Conversely, after full life-long exposure there was no apparent stimulation of VTG in the F_1_ adults, whereas short-term exposures strongly stimulated VTG in the parental F_0_ generation at similar doses. These data have important implications for bodies devising effective methodologies for the diagnosis of the ecologic risk from endocrine disruptors and for those devising laboratory testing strategies for hazard assessment. Because it is the impacts at the population level that are ultimately of most concern, special care must be taken when interpreting the results from either short-term testing procedures or from biomarkers such as VTG. Although integrative long-term multigenerational studies are expensive and time consuming, the mechanistic information they produce is invaluable if we want to understand both the potential population consequences of endocrine disruption and the mode of action that leads to reproductive failure. Although these long-term studies cannot be repeated for every potential EDC, the mechanistic knowledge gained should be integrated into the design of shorter testing methodologies and new mechanistic approaches such as toxicogenomics, to allow for more accurate predictions of the population level consequences.

The value of an integrative approach can be clearly seen from the data obtained in our male replacement studies. A differential in relative sensitivity to disruption between gonadal and behavioral sexual differentiation had the consequence that male-pattern behavior was more robust to the effects of EE_2_ exposure than was gonadal development, where exposure caused complete male infertility. If behavior is considered in isolation, then a lower impact on behavior may seem to have fewer population consequences; when behavior is considered in combination with infertility, it may actually increase the risk of reproductive failure in natural populations. We suggest that reproductive behavior plays a pivotal role in how chemically induced reproductive dysfunction(s) act to affect reproductive success and/or genetic integrity of populations. Moreover, there exists the possibility of other important interactions between endocrine disruption and mating systems that could strongly influence the population-level impact of reduced fertility. Sexual selection and mate choice strategies are complex, and endocrine disruption may interact or interfere with these in both negative and positive ways. Factors that determine the choice of a male by the female could, for example, be exaggerated in males with reduced fertility, giving these fish an even greater potential for interfering with the natural mating process and further increasing the impact of endocrine disruption on reproductive success. We suggest that further studies should examine this previously overlooked area.
